# Not All Scale-Free Networks Are Born Equal: The Role of the Seed Graph in PPI Network Evolution

**DOI:** 10.1371/journal.pcbi.0030118

**Published:** 2007-07-06

**Authors:** Fereydoun Hormozdiari, Petra Berenbrink, Nataša Pržulj, S. Cenk Sahinalp

**Affiliations:** 1 School of Computing Science, Simon Fraser University, Burnaby, British Columbia, Canada; 2 Department of Computer Science, University of California Irvine, California, United States of America; Yale University, United States of America

## Abstract

The (asymptotic) degree distributions of the best-known “scale-free” network models are all similar and are independent of the seed graph used; hence, it has been tempting to assume that networks generated by these models are generally similar. In this paper, we observe that several key topological features of such networks depend heavily on the specific model and the seed graph used. Furthermore, we show that starting with the “right” seed graph (typically a dense subgraph of the protein–protein interaction network analyzed), the *duplication model* captures many topological features of publicly available protein–protein interaction networks very well.

## Author Summary

The interactions among proteins in an organism can be represented as a protein–protein interaction (PPI) network, where each protein is represented with a node, and each interaction is represented with an edge between two nodes. As PPI networks of several model organisms become available, their topological features attract considerable attention. It is believed that the available PPI networks are (1) “small-world” networks, and (2) their degree distribution is in the form of a “power law.” In other words, (1) it is possible to reach from a protein to any other protein in only a small (approximately six) number of hops, and (2) although most proteins have only a few interactions (one or two), there are a few proteins with many more interactions (200 or more) and that act as “hubs.” It has thus been tempting to develop simple mathematical network generators with topological features similar to those of the available PPI networks. One such model, the “duplication model,” is based on Ohno's model of genome growth. It starts with a small “seed network” and grows by “duplicating” one of the existing nodes at a time, with an identical set of interactions; a randomly selected subset of these interactions is then deleted, and a few new interactions are added at random. It has been mathematically proven that the duplication model provides a small-world network and also has a power-law degree distribution. What we show in this paper is that by choosing the “right” seed network, many other topological features of the available PPI networks can be captured by the duplication model. The right seed network in this case turns out to include two sizable “cliques” (subnetworks where all node pairs are connected) with many interactions in between. In this paper, we also consider the preferential attachment model, which again grows by adding to a seed network one node at a time and connecting the new node to every other node with probability proportional to the existing degree of the second node. Because the preferential attachment model also provides a small-world network and has a power-law degree distribution, it has been considered equivalent to the duplication model. We show that the two models are vastly different in terms of other topological features we consider, and the preferential attachment model cannot capture some key features of the available PPI networks.

## Introduction

In the past few years, protein–protein interaction (PPI) networks of several organisms have been derived and made publicly available. Some of these networks have interesting topological properties (e.g., the degree distribution of the yeast PPI network is heavy-tailed; that is, there are a few nodes with many connections). It has been argued that the degree distribution of these networks are in the form of a *power law* [1,2] (some recent works challenge this by attributing the power law–like behavior to sampling issues, experimental errors, or statistical mistakes [3–7]). Since well-known random graph models also have power-law degree distributions [8–10], it has been tempting to investigate whether these models agree with other topological features of the PPI networks.

There are two well-known models that provide power-law degree distributions [11–13]. The *preferential attachment* model [9,14] was introduced to emulate the growth of naturally occurring networks such as the web graph; unfortunately, it is not biologically well-motivated for modeling PPI networks. The *duplication model,* on the other hand [15–17], is inspired by Ohno's hypothesis on genome growth [18] by duplication. Both models are iterative in the sense that they start with a *seed graph* and grow the network in a sequence of steps.

The degree distribution is commonly used to test whether two given networks are similar or not. However, networks with identical degree distributions can have very different topologies (e.g., consider an infinite 2-D grid versus a collection of cliques of five nodes; in both cases, all nodes have a degree of four). Furthermore, it was observed in [3] that given two networks with substantially different initial degree distributions, a partial (random) sample from those networks might give subnetworks with very similar degree distributions. Thus, the degree distribution cannot be used as a sole measure of topological similarity.

In the recent literature, two additional measures have been used to compare PPI networks with random network models. The first such measure is based on the *k-hop reachability.* The 1-hop reachability of a node is simply its degree (i.e., the number of its neighbors). The *k*-hop reachability of a node is the number of distinct nodes it can reach via a path of ≤*k* edges. The *k*-hop reachability of all nodes whose degree is λ is the average *k*-hop reachability of these nodes. Thus, the *k*-hop reachability (for *k* = 2,3,...) of nodes as a function of their degree can be used to compare network topologies. An earlier comparison of the *k*-hop reachability of the yeast network with networks generated by certain duplication models concluded that the two network topologies are quite different [19]. The second similarity measure is based on the *graphlet distribution.* Graphlets are small subgraphs such as triangles, stars, or cliques. In [4] it was noted that certain “scale-free” networks are quite different from the yeast PPI network with respect to the graphlet distribution. This observation, in combination with that on the *k*-hop degree distribution, seems to suggest that the known PPI networks may not be scale-free, and that existing scale-free network models may not capture the topological properties of the PPI networks.

There are other topological measures that have been commonly used in comparing social networks, etc., but not PPI networks. Two well-known examples are the *betweenness* distribution and the *closeness* distribution [20]. Betweenness of a node *v* is the number of shortest paths between any pair of nodes *u* and *w* that pass through *v*, normalized by the total number of such paths. Closeness of *v* is the inverse of the total distance of *v* to all other nodes *u*. Thus, one can use betweenness and the closeness distributions, which respectively depict the number of nodes within a certain range of betweenness and closeness values that can be used to compare network topologies.

## Results/Discussion

As mentioned above, scale-free network generation models such as the preferential attachment model and the duplication model can have very similar degree distributions under appropriate choice of parameters. (See Materials and Methods for exact definitions for the two network generation models.) Moreover, the degree distribution of these models converge to a power-law degree distribution whose shape is determined solely by the edge deletion and edge insertion probabilities, and not by the initial “seed” graph [11]. Hence, it has been tempting to assume that networks generated by these models are similar in general; moreover, the effect of the seed graph in shaping the topologies of these networks has largely been ignored in recent literature.

We start with the observation that two networks with very similar degree distributions may have very different topologies. For example, a network generated by the preferential attachment and another generated by the duplication model may have very different *k*-hop reachability, graphlet, betweenness, and closeness distributions while having almost identical degree distributions.

**Figure 1:**
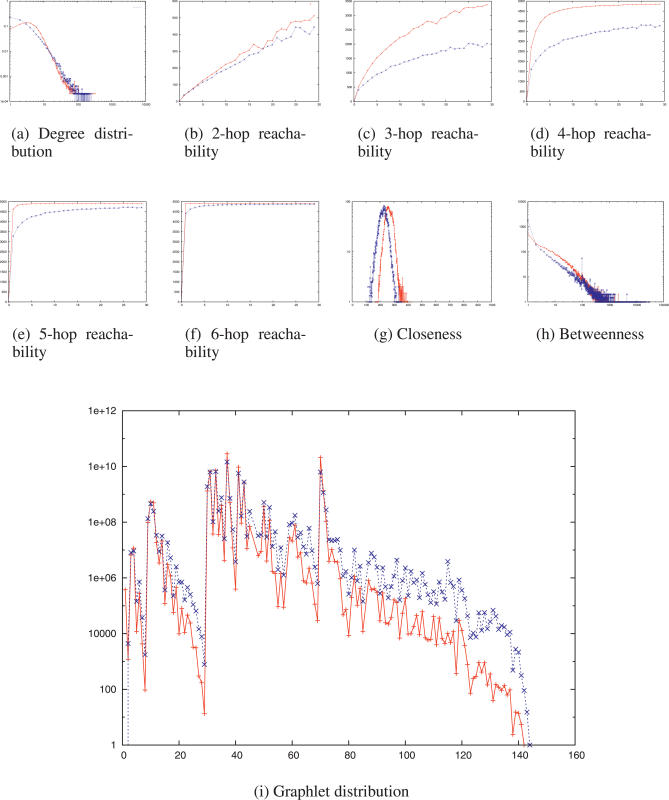
A Comparison of the Degree Distribution, *k*-Hop Reachability, Graphlet, Closeness, and Betweenness Distributions of the Preferential Attachment Model (Red) and the Duplication Model (Blue)

Figure 1 depicts the degree distribution, *k*-hop reachability, and graphlet frequency of the duplication model and the preferential attachment model with 4,902 nodes (as per the yeast PPI network [21]). Both models start with identical seed graphs; we set *r* = 0.12, *p* = 0.365 (the two key parameters of the duplication model), and *c* = 7 (the single key parameter of the preferential attachment model) so that the average degree of nodes in both models is seven (again as per the yeast PPI network [21]). Figure 1 compares the *k*-hop reachability achieved by the two models for *k* > 1. As can be seen, the *k*-hop reachability is quite different, especially for *k* = 3,4. Figure 1 also shows how the graphlet distributions differ, especially for dense graphlets (e.g., graphlets 17–29 and 85–145). In terms of betweenness and closeness, there are some differences as well.

**Figure 2:**
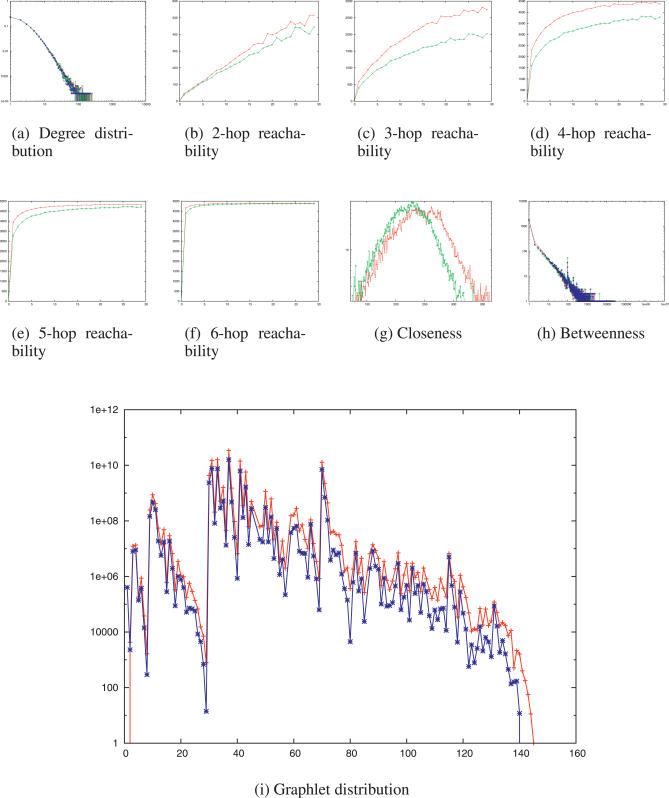
The Effect of the Seed Graph on the Degree Distribution, *k*-Hop Reachability, Graphlet, Closeness, and Betweenness Distributions of the Duplication Model Each color (red, blue, green) depicts the behavior of a network with a particular seed graph. The parameters *p* and *r* are identical in all three models.

We now show that the seed graph has a role in characterizing the topology of the duplication model. Figure 2 depicts how various topological features of the duplication model with fixed parameters (*p* = 0.365 and *r* = 0.12) vary as the seed graph changes. The first seed graph (red) is obtained by highly connecting two cliques of ten and seven nodes, respectively, by several random edges. To reduce the average degree, some additional nodes were generated and randomly connected to one of the cliques. The second seed graph (blue) is obtained by enriching a ring of 17 nodes by random connections so as to make the average degree match that of the first seed graph. The third seed graph (green) is formed by sparsely connecting two cliques of ten and seven nodes, respectively, with some added nodes randomly connected to one of the cliques.

All three networks were grown until all had 4,902 nodes as per the yeast PPI network [21]. (We depict the “average behavior” of five independent runs of each of the models.) It can be observed that although all of them have very similar degree distributions, their graphlet distributions may be quite different, especially for dense graphlets. Figure 2 also compares the *k*-hop reachability, closeness, and betweenness distributions. As can be seen, the *k*-hop reachability and the closeness distribution can vary considerably. Note that both the graphlet and the closeness distributions are in logarithmic scale, and seemingly small variations in the figure may imply several factors of magnitude of a difference between the two distributions.

The key question we aim to address in this paper is the following. If the seed selection has such an impact in shaping the topology of the network generated by the duplication model, is it possible to select the “right” seed graph so that all interesting topological features of the PPI networks in question can be captured? Also, is there a systematic way to determine a subgraph of a PPI network that can provide a good seed graph?

We answer the above questions positively by demonstrating that the duplication model applied on the right seed graph can result in a network that accurately captures all key features of the PPI networks we considered.

The PPI networks we consider in this study include (the largest connected component of) the complete Database of Interacting Proteins (DIP) yeast PPI network [21] with 4,902 proteins and 17,200 edges (as of July 2006) as well as the smaller but more accurate core yeast network from the DIP [22]. We also tested the lesser-developed DIP worm network [21]. (See Materials and Methods for a detailed description of these networks.) As will be demonstrated, we were able to closely approximate all the interesting topological features of these networks via the duplication model using specific seed graphs that largely exist as a subgraph in the corresponding PPI network.

A crucial observation toward obtaining the right seed graph is that the duplication model is unlikely to generate “large” cliques (a set of nodes which are fully connected). Notice that the only way to produce a clique of size *h* through the duplication model is starting with a clique of size *h* − 1, duplicating one of its nodes, and making sure that none of the new node's edges that are connected to the clique are deleted. The probability of this happening is negligible for large values of *h*.

The size of the maximum clique in the yeast PPI network is ten nodes. In our experiments with the duplication model, even if we started with a seed graph that included a clique of nine nodes (but not ten), the chances that we ended up with a clique of ten nodes (in <5,000 steps) turned out to be negligible. Thus, the seed graph *has* to include a clique of ten nodes.

We enriched the seed graph by adding to the clique of ten nodes another (independent) clique of seven nodes that is present in the yeast PPI network. We also included the edges between the two cliques and some additional nodes so that the normalized degree distribution of the yeast PPI network would be similar to that of the seed graph. The total number of nodes in the resulting seed graph was 50.

**Figure 3:**
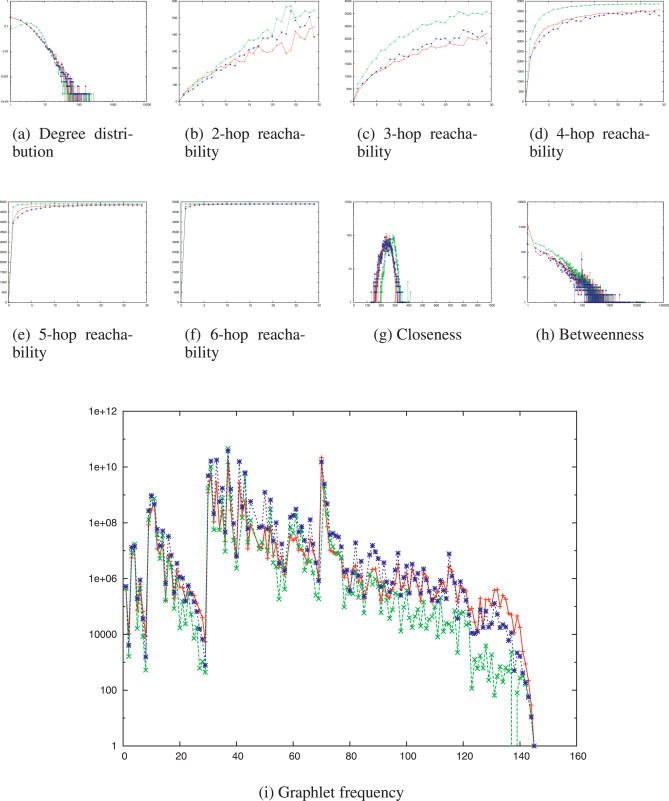
The Degree Distribution, the *k*-Hop Reachability, the Graphlet, Closeness, and Betweenness Distributions of the Yeast PPI Network (Red), Duplication Model (Blue), and Preferential Attachment Model (Green)

As mentioned before, there are two key parameters associated with the duplication model: *p*, the edge maintenance probability; and *r*, the edge insertion probability. These two parameters alone determine the (asymptotic) degree distribution and the average degree of the generated network. We chose *p* = 0.365 and *r* = 0.12 so that the degree distribution of the duplication model matches that of the yeast PPI network (see Methods and Materials for the exact mathematical expressions for *p* and *r*). Also, for the preferential attachment model, we choose the value *c* = 7 so that the average degree of the graph created using preferential attachment would be equal to that of the yeast PPI network. We used the duplication model and preferential attachment model described above to generate a network with 4,902 nodes. The resulting networks are compared with the yeast PPI network in terms of the *k*-hop reachability, the graphlet, betweenness, and closeness distributions in Figure 3. Under all these measures, the yeast PPI network is very similar to the network produced by the duplication model (and not similar to the network produced by the preferential attachment model). In fact, the duplication model approximates both the *k*-hop degree distribution and the graphlet distribution of the yeast network much better than the random graph models described earlier in the literature ([4] and [19])—which were specifically devised to capture the respective features of the yeast PPI network.

**Figure 4:**
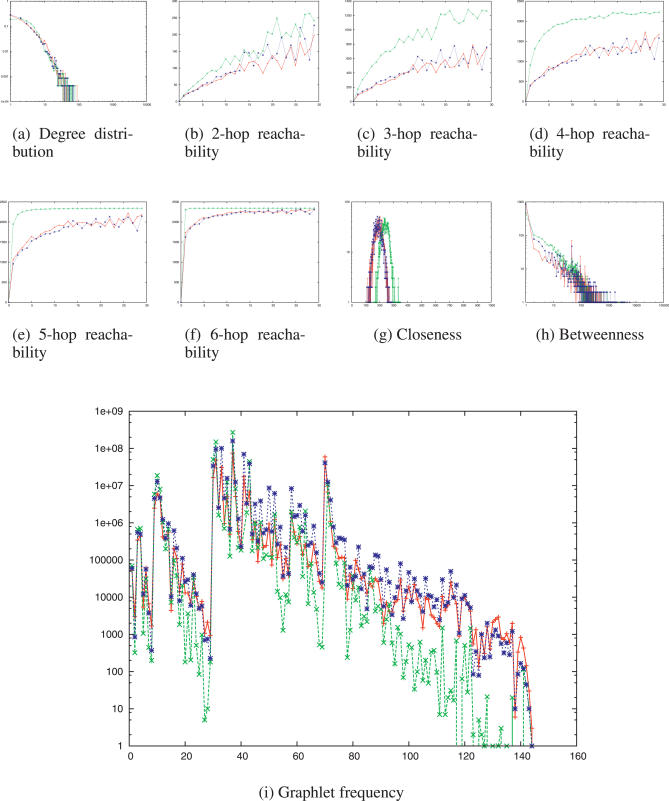
The Topological Properties of the Duplication Model (Blue) and Preferential Attachment Model (Green) Compared with That of the CORE Yeast PPI Network (Red) The degree distribution, the *k*-hop reachability, graphlet, closeness, and betweenness distributions of both networks are shown.

Another evidence of the power of the duplication model in capturing the topological features of available PPI networks is through comparing the duplication model with the main component of the core subset of the yeast network. The core subset contains the pairs of interacting proteins identified in the yeast that were validated according to the criteria described in [22]. It involves 2,345 nodes and 5,609 edges. The values of *r* and *p* were set to *r* = 0.12, *p* = 0.322 as prescribed by the average degree formula *a* = 2*r* / (1− *P_S_* − 2*p*) and the fact that *P_S_* is a function of *r* and *p* (see the next section for explanation). The seed graph we used was very similar to that used for the complete yeast network. Also, for the preferential attachment model, we set a value *c* = 4.8 so that the network generated using the model has the same average degree as the CORE yeast PPI network. The results are shown in Figure 4.

Although the yeast PPI network is the most reliable PPI network available, it is still far from completion. Following up on [3], we also considered the effect of sampling errors on the duplication model with respect to all the topological features used.

In order to emulate the effect of sampling and thus the (potential) presence of false negatives in the yeast PPI network, we used the duplication model to generate larger networks than the available ones and applied the sampling strategy proposed in [3] to “shrink” them to the size of the available networks. The sampling strategy of [3] involves two parameters: the bait sampling probability (the probability that a node is kept in the network during sampling) and the edge sampling probability (the probability that an edge of a bait is kept in the network). We demonstrate the effect of sampling as per [3] on the emulation of both the full yeast and the CORE yeast PPI networks below.

**Figure 5:**
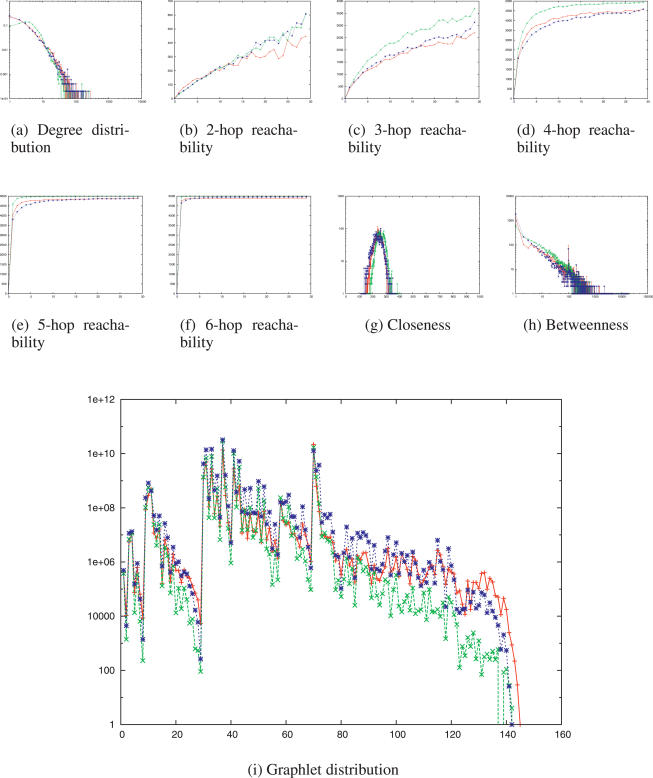
Comparison of Duplication (Blue) and Preferential Attachment (Green) with 70% Bait and 70% Edge Coverage against the Yeast PPI Network (Red)

We used a bait sampling probability and an edge sampling probability of 0.7 each (resulting in 70% “bait coverage” and again 70% “edge coverage”) for our emulation of the full yeast PPI network. A comparison of the features of the resulting network with that of the full yeast PPI network is given in Figure 5.

We then used a bait sampling probability and edge sampling probability of 0.5 each for emulating the core yeast PPI network (resulting in 50% “bait coverage” and 50% “edge coverage”).

**Figure 6:**
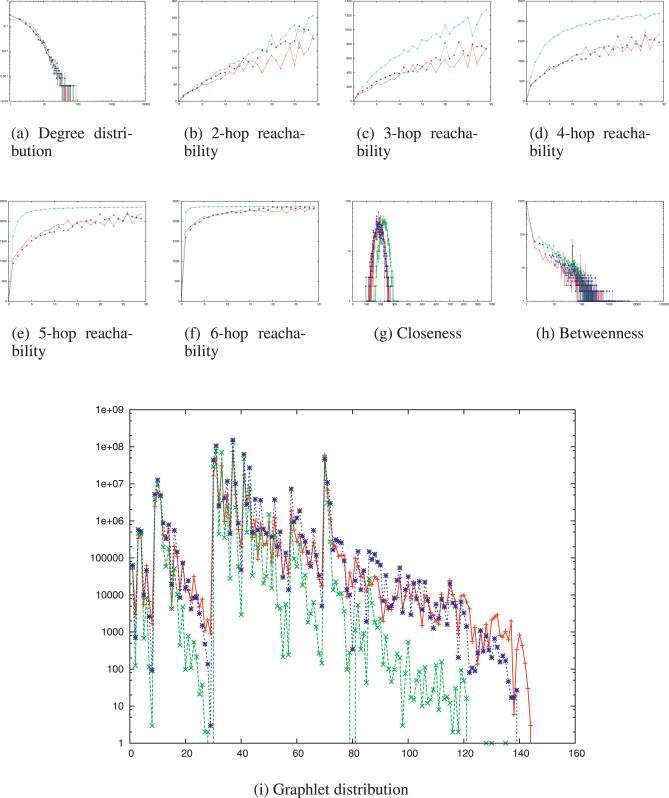
Comparison of Duplication (Blue) and Preferential Attachment (Green) with 50% Bait and 50% Edge Coverage against the CORE Yeast PPI Network (Red)

A comparison of the core yeast PPI network against the resulting network is given in Figure 6. As can be seen, the topological features of both the full yeast PPI network and the core yeast PPI can still be closely captured by the networks obtained via the duplication model, which have been subject to sampling errors.

The seed graphs used in both tests involving sampling are identical to those used in the tests that do not involve sampling. Uniform sampling reduces the size of the maximum clique in the resulting networks significantly, as can be seen at the tail end of the graphlet distributions. In reality, the sampling errors are not uniform. Very dense subnetworks such as cliques are better covered by both the full yeast network and the core yeast network of the DIP. It is interesting to note that although the core yeast network has only 5,609 edges in comparison to the full yeast network's 17,200 edges, the maximum clique size in the former is nine nodes, whereas it is ten nodes in the latter.

## Materials and Methods

Here we describe in detail the PPI network data we used in our analysis. We also formally describe the network generation models we used, namely the preferential attachment model and a modified version of the duplication model (which does not generate too many singletons). We show how to set the parameters of the modified duplication model so that it achieves a given degree distribution (e.g., that of the yeast PPI network) as well. We also describe in detail the topological features we use for comparing two networks.

### PPI network data.

Perhaps the best-known PPI network database is DIP [21], which includes the Saccharomyces cerevisiae (yeast) PPI network (the best-developed PPI network available, with 4,902 proteins and 17,200 interactions). DIP also includes a more accurate but much smaller core yeast network (2,345 proteins and 5,609 interactions) [22]. Our results are mainly on these two networks. Although there are other PPI networks available through DIP [23] (e.g., those of the fruit fly, human, and mouse) as well as through BIND [24], IntAct [25], and MINT [26] databases, they are not sufficiently well-developed to perform a conclusive analysis. For comparison purposes, we also provide results on the DIP Caenorhabditis elegans (worm) PPI network (which includes 2,387 proteins and 3,825 interactions) as Text S1.

### Network generation models.

The two network models we study here, namely the preferential attachment model and the duplication model, both start with a small seed graph and create an additional node in each iteration as described below. For notational convenience, let *G*(*t*) = (*V*(*t*), *E*(*t*)) be the network at the end of time step *t*, where *V*(*t*) is the set of nodes and *E*(*t*) is the set of edges/connections. Let *v_t_* be the node generated in time step *t*. Given a node *v_t_*, we denote its degree at the end of time step *t* by *d_t_*(*v_t_*).

The preferential attachment model (as analyzed in [9,11,14,27]) generates a network as follows. In iteration *t* a new node *v_t_* is generated and is connected to every other node *v_t_* in the network independently with probability 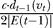 . Here, *c* is the average degree of a node in *G*.

The duplication model (as analyzed in [15–17]), in contrast, generates a network as follows. In iteration *t*, an existing node *v_t_* of *G*(*t* − 1) is picked uniformly at random and “duplicated” (i.e., an exact copy of *v_t_* as *v_t_* is generated). The edge set of *v_t_* is then updated: first, each existing edge of *v_t_* is deleted independently with probability (1 − *p*); then each node *v_t_* not connected to *v_t_* is connected to *v_t_* independently with probability *r* / |*V*(*t*)|. Here, *p* and *r* are user-defined parameters. Note that it is possible to maintain a constant average degree (*a*) throughout the generation of the network by setting *r* = (1/2 − *p*) · *a*.

As mentioned earlier, the degree distributions of both the preferential attachment model and the duplication model asymptotically approach a power law [9,11,12,14]. More specifically, the frequency of nodes with degree *d* is proportional to *d^−b^*, where *b* is a constant typically between 2 and 3. The value of *b* is solely determined (asymptotically) by the values of *p* and *r* in the duplication model or the value of *c* in the preferential attachment model.

Both the preferential attachment and the duplication model produce many *singletons* [13] (i.e., nodes that are not connected to any other node). (For example, in the duplication model where *r* = 0, *p* = 1/2, the proportion of singletons asymptotically approaches 1.) In contrast, the number of singletons in known PPI networks is very small (this is not surprising, as genes with “no functionality” are not maintained by evolution).

To avoid the generation of singletons, it is possible to use a slightly modified duplication model that deletes each singleton node as soon as it is generated. This modified duplication model has also been shown to achieve a power-law degree distribution [13]. However, it is not known which values of *p* and *r* ensure that the expected average degree can be set to a desired value and is kept fixed through all iterations. In this paper, we derive conditions on *p* and *r* that are necessary for having a constant expected degree. We later use these conditions so that the modified duplication model can approximate the degree distribution of the yeast PPI network as tightly as possible.

### Network comparison methods.

Perhaps the ultimate way to test whether two networks are topologically similar or not is through the use of graph isomorphism as described below. Unfortunately, graph isomorphism and approximate graph isomorphism are computationally hard problems. Thus, it is very common to use some of the topological features of networks as a basis of checking their similarity. In this paper, we focus on five such features: the degree distribution, the *k*-hop reachability, the graphlet frequency, the betweenness distribution, and the closeness distribution.

*Graph isomorphism.* Two networks, *G* and *G′*, are called *isomorphic* if there exists a bijective mapping *F* from each node of *G* to a distinct node in *G′*, such that two nodes *v* and *w* are connected in *G* if and only if *F*(*v*) and *F*(*w*) are connected. *G* and *G′* are called approximately isomorphic if by removing a “small” number of nodes and edges from *G* and *G′*, they could be made isomorphic. Ideally, a random graph model that aims to emulate the growth of a PPI network should produce a network that is approximately isomorphic to the PPI network under investigation. Unfortunately, the problem of approximate isomorphism is NP-complete (through a trivial reduction from subgraph isomorphism—a known NP-complete problem); thus, this measure cannot be used to practically test similarity of two networks.

*k-hop reachability.* Let *V*(*i*) denote the set of nodes in *G* whose degree is *i*. Given a node *v*, denote by *d*(*v*,*k*) its *k*-hop degree (i.e., the number of distinct nodes it can reach in ≤*k*-hops). Now we define *f*(*i*,*k*), the *k*-hop reachability of *V*(*i*), as

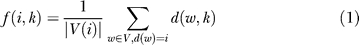



Note that *f*(*i*,*k*) is the “average” number of distinct nodes a node with degree *i* can reach in *k*-hops (e.g., *f*(*i*,1) = *i* by definition).

*Graphlet frequency.* The graphlet frequency was introduced in [4] to compare the topological structure of networks. A graphlet is a small connected induced subgraph of a large graph (e.g., a triangle or a clique). The *graphlet count* of a given graphlet *g* with *r* nodes in a given graph *G* = (*V*,*E*) is defined as the number of distinct subsets of *V* (with *r* nodes) whose induced subgraphs in *G* are isomorphic to *g*. In this paper, we consider all 141 possible graphlets/subgraph topologies with three, four, five, and six nodes. In addition, we consider cliques of sizes seven, eight, nine, and ten. We enumerate these graphlets as shown in the final figure in Text S2.

*Betweenness distribution.* The betweenness of a fixed node of a network measures the extent to which a particular point lies “between” point pairs in the network *G* = (*V*,*E*). The formal definition of betweenness is as follows. Let s*_x,y_* be the number of the shortest path from *x* ∈ *V* to *y* ∈ *V* for all pairs *x*,*y* ∈ *V*. (Note that s*_x,y_* = s*_y,x_* in undirected graphs.) Let s*_x,y_*(*v*) be the number of shortest paths from *x* ∈ *V* to *y* ∈ *V* which go through node *v*. The betweenness *Bet*(*v*) of node *v* is now defined as

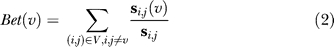



*Closeness.* For all *x*,*y* ∈ *V*, we define *d_x,y_* as the length of the shortest path between *x* and *y*. The closeness of a node *v* ∈ *V* is defined as

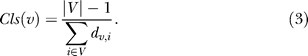
Thus, closeness of a node *v* is simply the inverse of the average distance of *v* to all other nodes in *G*.

**Figure 7:**
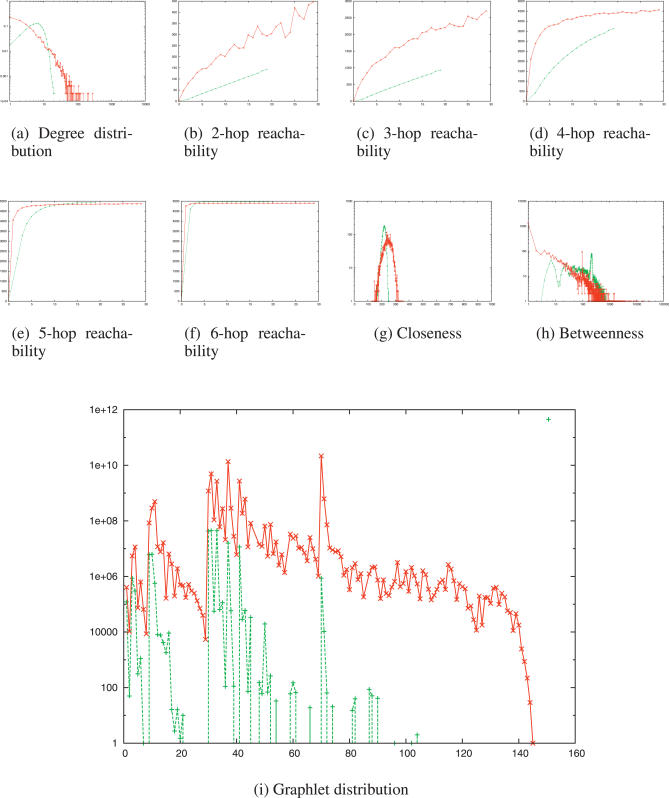
A Comparison of the Yeast PPI Network (Red) and (Five Independent Runs of) the Erdos–Renyi Random Graph Model (Green)

*The network comparison methods in use: The yeast PPI network versus the Erdos–Renyi random graph model.* The network features described above can be used to test whether a given random graph model can emulate an available PPI network. Here, we consider the standard Erdos*–*Renyi random graph model [28] in comparison to the yeast PPI network. As shown in Figure 7, each of the features we consider point to significant differences between yeast PPI (red) and (five independent runs of) the Erdos*–*Renyi (green) model.

### Determining the parameters of the modified duplication model.

In this section, we show how to determine the deletion probability 1 − *p* with respect to the insertion probability *r* so that the expected average degree of the network can be set to any given value. For this, we make the assumption that the degree frequency distribution and the average degree of nodes are fixed asymptotically once the values of *p* and *r* are determined. Let *G*(*t*) = (*V*(*t*), *E*(*t*)) be the network generated by the modified duplication model and let *n*(*t*) = |*V*(*t*)| and *e*(*t*) = |*E*(*t*)|. Also, let *n_k_*(*t*) be the number of nodes in time step *t* with degree *k* and *a*(*t*) be the average degree of nodes in *G*(*t*). Finally, let *P_k_*(*t*) = *n_k_*(*t*) / *n*(*t*), the frequency of nodes with degree *k* at time step *t*. We assume that *P_k_* *(t)* is asymptotically stable (i.e., *P_k_*(*t*) = *P_k_*(*t* + 1) for all 1 ≤ *k* ≤ *t* for sufficiently large values of *t*. In other words, we assume that *P_k_*(*t*) = *d_k_* for some fixed *d_k_*. By definition:

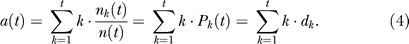



Now we can calculate the average degree *a*(*t* + 1) under the condition that degree frequency distribution is stable and *a*(*t*) = *a*, a constant.

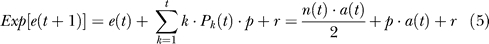



Let Pr*_s_*(*t*) be the probability that *v_t_*_+1_ ends up as a singleton.

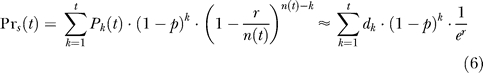



Since this probability does not depend on *t* asymptotically, we can set Pr*_s_*(*t*) = Pr*_s_*. Now we can calculate the expected number of nodes and the expected number of edges in step *t* + 1.

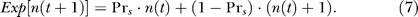


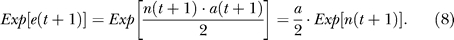


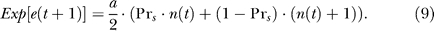



Comparing the above equation with the first equation for *Exp*[*e*(*t* + 1)], we get

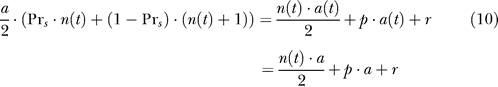



Solving Equation 10 results in *a* = 2*r* / (1 − Pr*_s_* − 2*p*), where Pr*_s_* is a function of *p*, *r*, and *d_k_* only.

The discussion above demonstrates that the two key parameters *p* and *r* of the (modified) duplication model are determined by the degree distribution and the average degree of the PPI network we would like to emulate.

## Supporting Information

Text S1 Worm PPI Network (99 KB PDF)

Text S2 The Enumeration Used for Graphlet Distributions (122 KB PDF)

## Abbreviations

DIP: Database of Interacting Proteins
PPI: protein–protein interaction
