# Supplementary material for: Not All Scale-Free Networks Are Born Equal: The Role of the Seed Graph in PPI Network Evolution
Source: PLoS Comput Biol. 2007 Jul 6;3(7):e118. doi: 10.1371/journal.pcbi.0030118 (PMC1913096; doi:10.1371/journal.pcbi.0030118)
Supplement: Supplementary file 1 [file pcbi.0030118.sd001.pdf]

1 Worm PPI Network

Here we compare the duplication model (as well as the preferential attachment model) with the DIP Worm PPI network. This network is much less developed than the Yeast network with only 2387 nodes and 3825 edges. The values of r and p we used for this network are $r = 0.12$, $p = 0.322$. The seed graph we used was again very similar to that used for the Yeast network. The c value for preferential attachment was set to 3.2 to capture the average degree. The comparative results are shown in Figure 1.

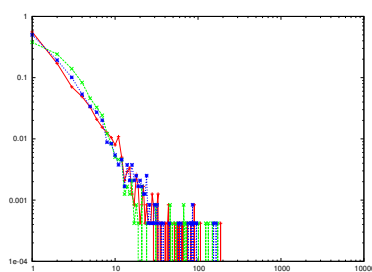

(a) Degree distribution

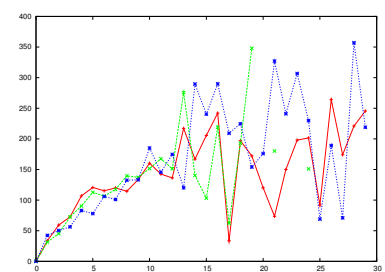

(b) 2-hop reachability

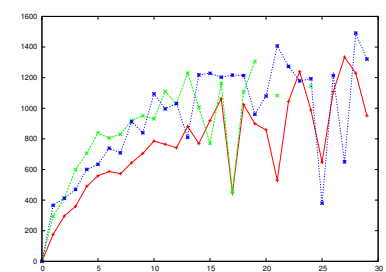

(c) 3-hop reachability

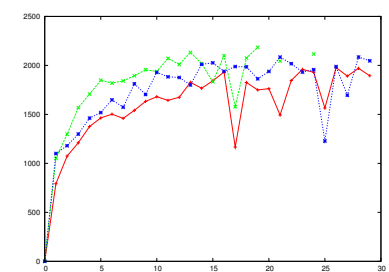

(d) 4-hop reachability

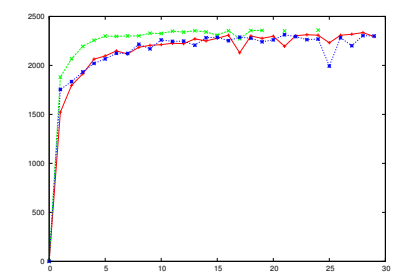

(e) 5-hop reachability

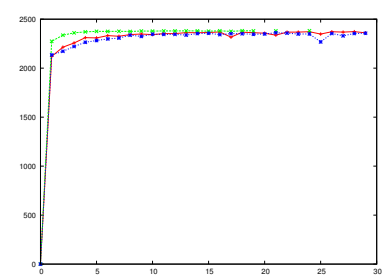

(f) 6-hop reachability

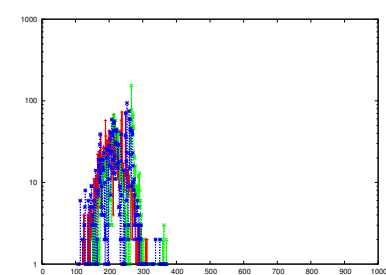

(g) Closeness

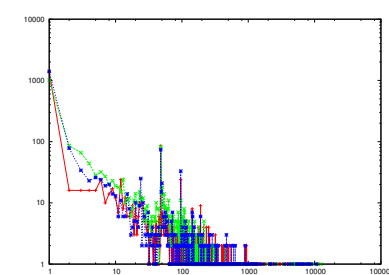

(h) Between-ness

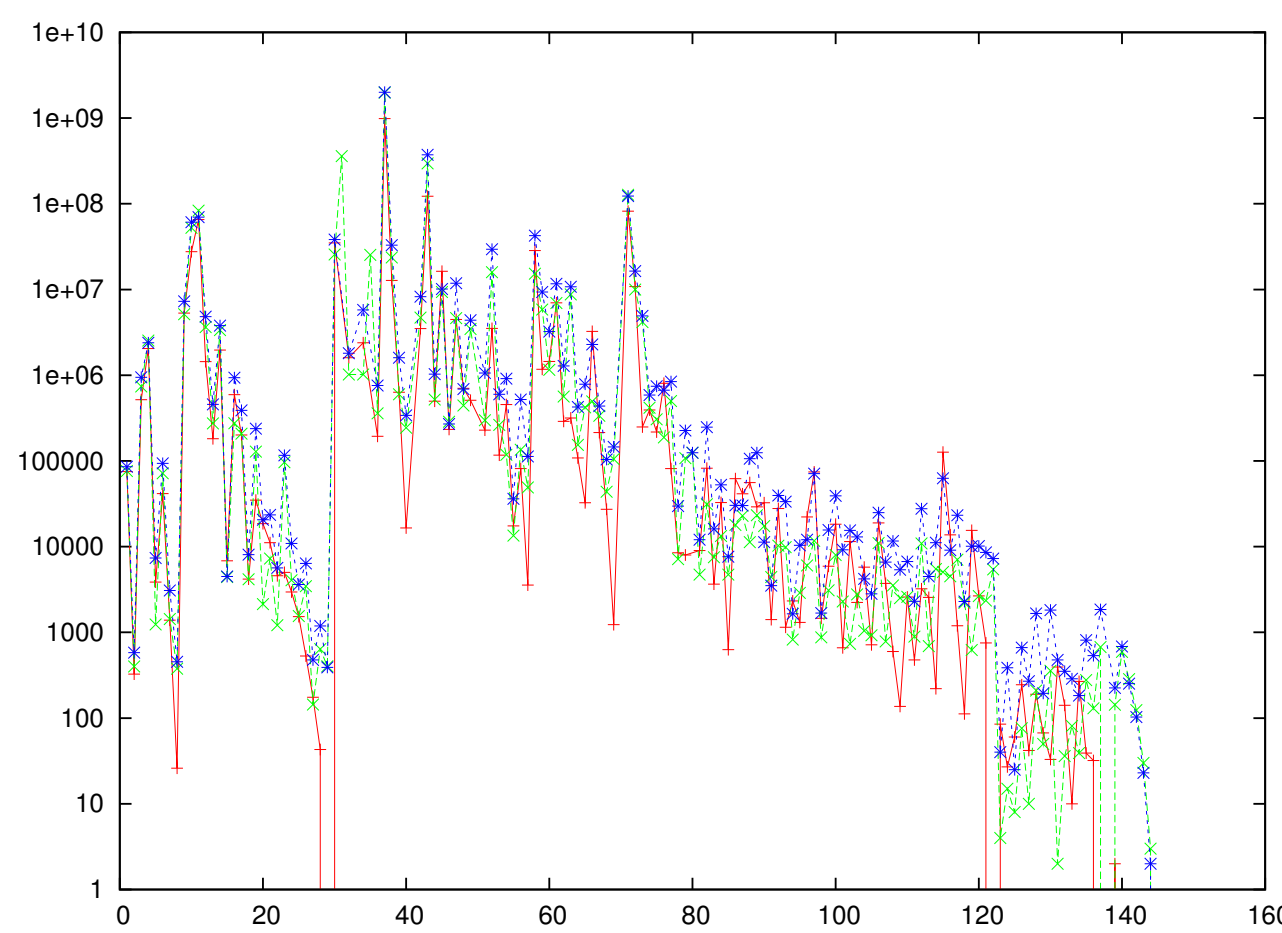

(i) Graphlet frequency

Figure 1: The topological properties of the duplication model (Blue) and preferential attachment model (Green) compared to that of the *C.Elegans* (Worm) network (Red). The degree distribution, the k-hop reachability, graphlet, betweenness and closeness distributions of both networks are shown.
