# Supplementary material for: Not All Scale-Free Networks Are Born Equal: The Role of the Seed Graph in PPI Network Evolution
Source: PLoS Comput Biol. 2007 Jul 6;3(7):e118. doi: 10.1371/journal.pcbi.0030118 (PMC1913096; doi:10.1371/journal.pcbi.0030118)
Supplement: Supplementary file 2 [file pcbi.0030118.sd002.pdf]

1 Graphlets

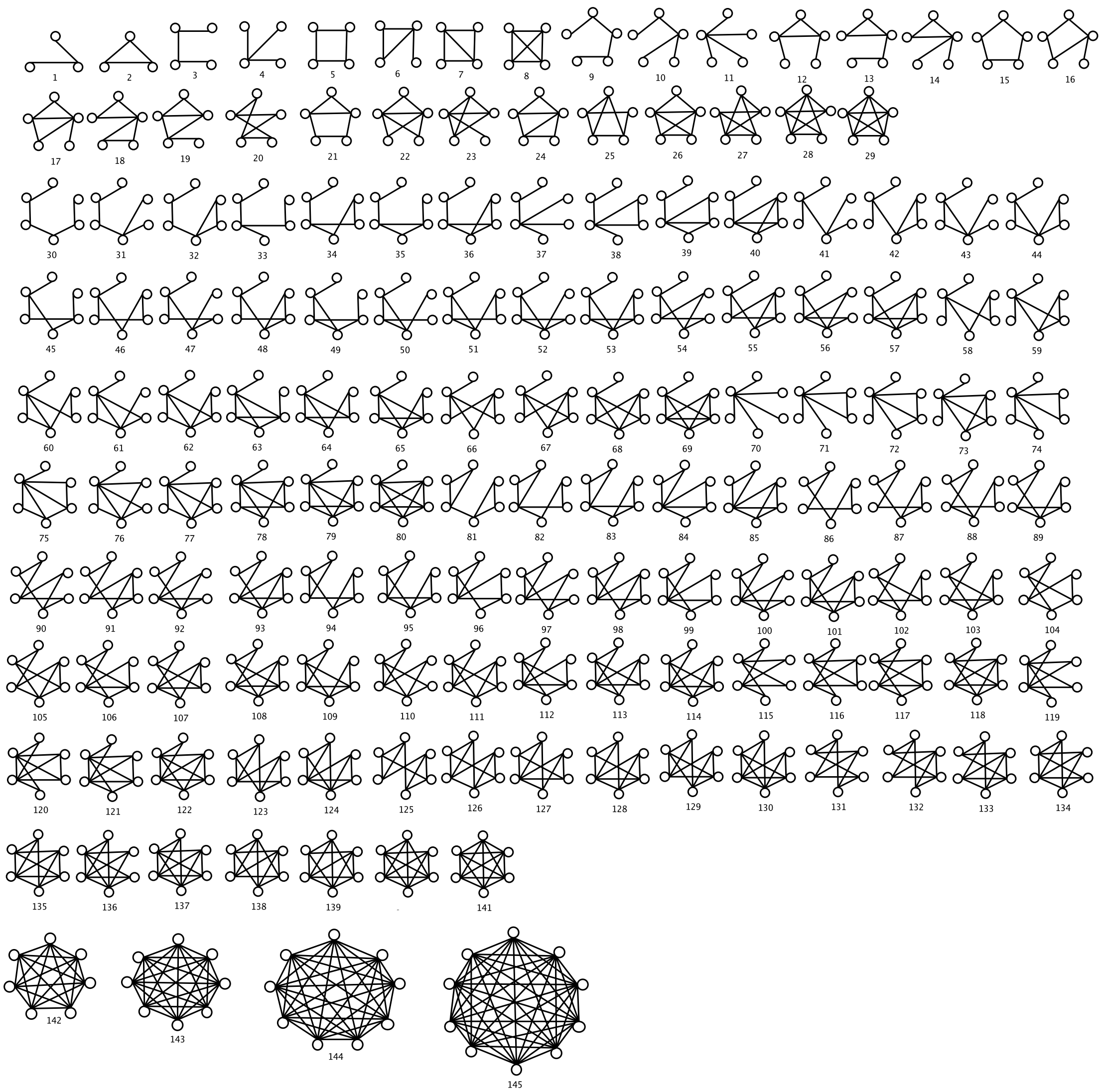

Figure 1: The enumeration used for graphlet distributions.
